# Small bowel Gastrointestinal Stromal Tumors can physiologically alter gut motility before causing mechanical obstruction

**DOI:** 10.1186/1477-7800-2-24

**Published:** 2005-10-26

**Authors:** Manish S Kothari, Vasilis Kosmoliaptsis, John Meyrick-Thomas

**Affiliations:** 1 Specialist Registrar, General Surgery, Charing Cross Hospital, London W6 8RF, UK; 2 Senior House Officer, General Surgery, Watford General Hospital, Watford, WD18 0HB, UK; 3 Consultant Surgeon, General Surgery, Watford General Hospital, Vicarage Road, Watford, WD18 0HB, UK

## Abstract

**Background:**

Gastro Intestinal Stromal Tumors (GISTs) are rare stromal neoplasms that represent the most common mesenchymal tumor of the G.I. tract, accounting for 5% of all sarcomas [1,2]. Originating from interstitial cells of Cajal, which are regulators of gut peristalsis, they are preferentially located in the stomach and the small intestine [3] and clinical presentation is variable, ranging from vague complaints to major G.I. bleeding. Surgical resection is the mainstay of treatment for patients with resectable GIST and 5-year survival ranges from 21% to 88% in different series depending on risk grading and completeness of surgical resection [4,5]. Imatinib mesylate, a tyrosine kinase inhibitor, provides an encouraging option for treating high risk GISTs.

**Case presentation:**

We report the case of a 62-year-old lady who had been diagnosed and being treated unsuccessfully for Irritable bowel syndrome for 11 years and eventually found to have an obstructing small bowel GIST.

**Conclusion:**

The symptoms from GIST may mimic those of irritable bowel syndrome. A physiological alteration in gut peristalsis resulting from neoplastic transformation of the interstitial cells of Cajal, is a hypothesis that could explain this presentation. An alternative diagnosis should be considered when treating patients with irritable bowel syndrome who fail to respond for a prolonged period.

## Case Report

A 69 year old lady presented 11 years ago with pain of mild to moderate severity on the right side of her abdomen. This pain was intermittent, described as 'no more than a dull ache' and associated with nausea, bloating and flatulence. She also suffered from constipation and experienced partial relief of her symptoms following defeacation. She reported worsening of her symptoms after a heavy meal, and experienced occasional bouts of steatorrhea. Physical examination revealed mild right-sided abdominal tenderness only.

Apart from the above she was a healthy lady without any co morbidity and no history of previous abdominal surgery. Initial investigations included an abdominal ultrasound scan (USS), a hepatobiliary iminodiacetic acid (HIDA) scan and an oesophago-gastro-duodenoscopy (OGD), which did not reveal any abnormality. Flexible sigmoidoscopy and colonoscopy were also unremarkable. Her pain was thought to be due to a slow transit colon and she was advised to take laxatives on a regular basis, which resulted in partial and temporary relief of her symptoms. Although her condition did not impact her lifestyle significantly, her symptoms never subsided completely and almost two years following initial presentation she underwent an endoscopic retrograde cholangiopancreatography (ERCP), a cream absorption test, wheat intolerance test and a CT scan of her abdomen. All of these did not reveal any abnormality and thus irritable bowel syndrome was thought to represent best her condition. She was also advised to take antispasmodics together with various combinations of laxatives and a course of Pancrelipase (Creon) tablets for presumed pancreatic insufficiency, which resulted in only temporary relief.

An opinion of a gastroenterology consultant physician was sought, resulting in another abdominal USS and OGD, as well as a barium follow through study which were all normal and thus she continued to take symptomatic treatment.

Finally, 11 years from presentation, the patient was admitted to hospital with acute small bowel obstruction and underwent a laparotomy within 24 hours of admission after adequate fluid and electrolyte replacement. At the operation a sizable mid-ileum, obstructing tumor was revealed, that was wedge resected with adequate gross margins followed by end to end hand sewn anastomosis. The rest of the laparotomy was unremarkable with no evidence of macroscopic spread of disease.

**Figure 1:**
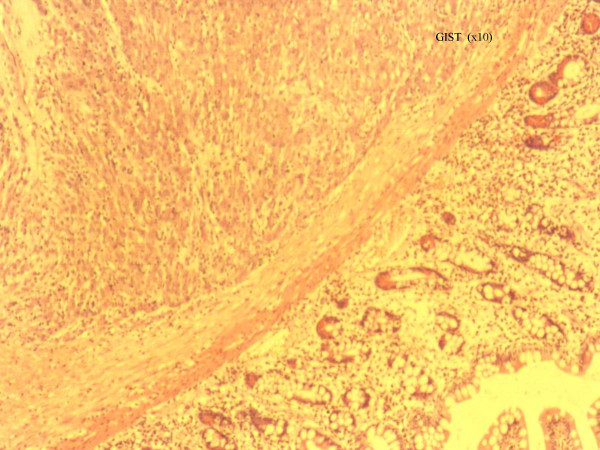
Histopathological appearance of the tumour under low and higher magnification respectively. The tumour consists of densely crowded spindle and polygonal cells with moderate nuclear pleomorphism. It has involved all the layers of the small bowel wall from mucosa outwards to the serosa.

**Figure 2:**
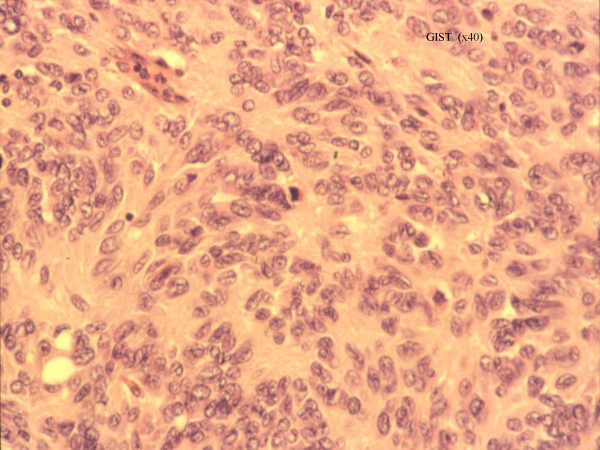
Histopathological appearance of the tumour under low and higher magnification respectively. The tumour consists of densely crowded spindle and polygonal cells with moderate nuclear pleomorphism. It has involved all the layers of the small bowel wall from mucosa outwards to the serosa.

**Figure 3:**
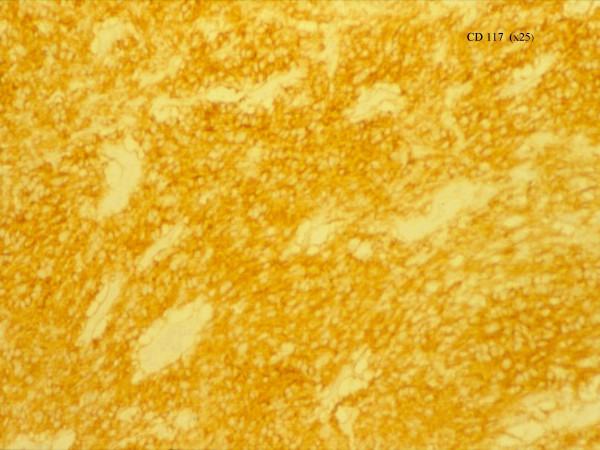
The tumour stained strongly for CD117 at immunostaining.

Histopathological analysis revealed a densely crowded spindle and polygonal cell tumour showing moderate nuclear pleomorphism (Figures 1 and 2) . No mitoses were found and the tumour spread into the mucosa and outwards into the serosa. There was no lymph nodal spread. Immunohistochemistry demonstrated positive staining for CD117 (cKit) (figure 3), and negative staining for broad range cytokeratins (MNF 116), CD34, desmin, S-100 and chromogranin. Although there were no mitoses, based on the size (80 × 40 × 50 mm), the tumour was classified as a GIST of intermediate malignant potential.

The patient made an uneventful recovery and remarkably, all her previous abdominal symptoms were completely resolved. A CT scan of the chest, abdomen and pelvis performed subsequently did not reveal the presence of metastatic disease. Her case was discussed in our multidisciplinary team meeting and it was decided not to proceed with any adjuvant treatment. At one year follow up our patient was a very happy lady completely free of her abdominal symptoms.

## Discussion

Although morphologically similar to other benign and malignant smooth muscle and neural stromal tumors, GIST constitutes a distinct group of rare gastrointestinal tract tumors that originate from the interstitial cells of Cajal [6]. The latter are regulators of gut peristalsis and normally express CD117, which is a product of the c-kit proto-oncogene that encodes a tyrosine kinase receptor, which regulates cellular proliferation in GISTs [1,6].

GISTs arise from the muscularis mucosa or muscularis propria layers and most exhibit an endophytic growth pattern, growing within the bowel lumen. The overlying mucosa is usually preserved but larger and more aggressive tumors tend to ulcerate through this. In up to one third of patients the tumor invades an adjacent organ. The vast majority of GISTs (up to 70%) arise in the stomach, with 20–30% originating in the small intestine and the remainder 10% occurring in the oesophagus, colon and rectum [1,3].

The clinical presentation is variable and depends on tumor size and anatomic site. Their submucosal location can produce local obstructive symptoms, particularly when arising in the oesophagus or the small intestine. Vague upper abdominal pain, fullness, GI bleeding, palpable mass are other modes of presentation whereas sometimes they are found incidentally during barium studies, endoscopy or abdominal scans performed for other reasons [1]. According to some authors, visceral obstruction is a rare occurrence even in the presence of extensive peritoneal metastatic disease [7].

Our patient had a very long course of vague symptoms consisting of abdominal pain, nausea, bloating and constipation, which were initially attributed to impaired gut motility as part of the irritable bowel syndrome. Her symptoms, however, failed to improve sufficiently to any treatment approach.

As mentioned above GIST's arise from the interstitial cells of Cajal, which play an important role in the regulation of gut peristalsis. Neoplastic transformation of these cells to a GIST could possibly result in alterations of the normal, local regulation of the gut motility. This hypothesis could, at least in part, explain our patient's symptoms especially at the initial stages of her presentation when the tumor would be too small to cause mechanical obstruction. More research into the physiology of the gut motility in relation to a GIST tumor would be needed to support or reject such a hypothesis.

## Conclusion

The occurrence of a GIST in the small bowel can present with vague symptoms, in our case symptoms thought to be from IBS for 11 years. We suggest that at least in the initial years our patients' symptoms may have been the result of functional alteration of gut peristalsis due to the increased number of CD117 positive cells in a slow growing GIST. That would suggest that GISTs can alter the motility of the G.I tract, even when the tumor is significantly small and thus difficult to detect on routine investigations. The symptoms may mimic those of irritable bowel syndrome and an alternative diagnosis should be considered when treating patients who fail to respond for a prolonged period. Resection of the tumour resulting in complete resolution of her symptoms strengthens the above suggestion. Although a diagnostic laparoscopy may not have helped detect the tumour when very small, it could have been considered at some stage during the years preceding her final presentation as acute intestinal obstruction.

## Competing interests

The author(s) declare that they have no competing interests.

## Authors' contributions

MSK has contributed towards conception, design, analysis and interpretation of data

VK has contributed towards conception, acquisition of data and preparation of the draft.

JMT has contributed towards revising the manuscript critically and has given final approval for the version to be published.
